# Study of the Psychometric Properties of the Spanish Version of the Measure of Moral Distress for Health Care Professionals (MMD-HP-SPA)

**DOI:** 10.3390/ijerph192315649

**Published:** 2022-11-25

**Authors:** Eloy Girela-López, Cristina M. Beltran-Aroca, Jaime Boceta-Osuna, Dolores Aguilera-Lopez, Alejandro Gomez-Carranza, Miguel García-Linares, Antonio Llergo-Muñoz, Manuel Romero-Saldaña

**Affiliations:** 1 Section of Legal and Forensic Medicine, Facultad de Medicina y Enfermería, Universidad de Córdoba, 14004 Cordoba, Spain; 2 Unidad de Cuidados Paliativos, Hospital Universitario Virgen Macarena, 41009 Sevilla, Spain; 3 Distrito Sanitario Córdoba-Guadalquivir, 14011 Cordoba, Spain; 4 Unidad de Cuidados Intensivos, Hospital Universitario Poniente, 04700 Almeria, Spain; 5 Equipo de Soporte Domiciliario de Cuidados Paliativos, Distrito Sevilla Norte-Aljarafe, 41008 Sevilla, Spain; 6 UGC Cuidados Paliativos, Hospital Universitario Reina Sofía, 14004 Cordoba, Spain; 7 Department of Nursing, Pharmacology and Physiotherapy, Facultad de Medicina y Enfermería, Universidad de Córdoba, 14004 Cordoba, Spain; 8 Grupo Asociado GA-16 Estilos de Vida, Innovación y Salud, Instituto Maimónides de Investigación Biomédica de Córdoba (IMIBIC), 14004 Cordoba, Spain

**Keywords:** moral distress, stress psychological, health personnel, psychometrics

## Abstract

Background: The early detection of moral distress requires a validated and reliable instrument. The aim of this study was to carry out an advanced analysis of the psychometric properties of the moral distress scale for health professionals (MMD-HP-SPA) by performing a validation of the construct and its internal and external reliability. Methods: We performed a multicentre cross-sectional study in health professionals belonging to the Andalusian public health system. Construct validity was performed by exploratory (*n* = 300) and confirmatory (*n* = 275) factor analysis (EFA/CFA) in different subgroups; we also analysed the internal consistency and temporal reliability of the scale. Results: 384 doctors and 191 nurses took part in the survey. The overall mean for moral distress was 128.5 (SD = 70.9), 95% CI [122.7–134.3], and it was higher in nurses at 140.5 (SD = 74.9) than in doctors at 122.5 (SD = 68.1), F = 8.37 *p* < 0.01. The EFA produced a model of five components which accounted for 54.8% of the variance of the model. The CFA achieved a goodness of fit of Chi^2^ = 972.4; AIC = 1144.3; RMSEA = 0.086; CFI = 0.844; TLI = 0.828; NFI = 0.785. Conclusions: The MMD-HP-SPA scale has solid construct validity, excellent internal consistency, optimal temporal reliability, and underlying dimensions which effectively explore the causes of moral distress in health professionals, thus guaranteeing its use in hospital and community settings.

## 1. Introduction

Moral distress is a major issue in routine clinical practice. Doctors and nurses in particular are exposed to witnessing or carrying out decisions or actions that contradict their fundamental moral values [1], as well as many other difficulties, such as long shifts, stress, and high levels of responsibility [2]. Its incidence has increased in health professionals around the world [1,3,4] due to work-related factors [5,6,7,8], personal factors [9], and as a result of the situations produced by the COVID-19 pandemic [10]. The current intense economic approach to contain costs and improve efficiency is not the primary goal of healthcare professionals. Economic rationality deprives physicians of the moral experience of medicine (restoring health and alleviating human suffering) that commits them. It is perhaps not surprising that physician burnout, suboptimal care, and diminished humanity are among the unintended outcomes of modern healthcare [11]. On the other hand, experiencing moral distress correlates with lower quality of care, in terms of patient safety and effectiveness of care [12], and some authors suggest that in the current climate moral distress is additionally shared by the patient [13].

Accurate, early detection using a validated and reliable instrument is of vital importance to develop an effective intervention strategy [14,15]. In the context of nursing work, Corley et al. [16] designed a 32-item moral distress scale (MDS), which was later revised and simplified to 21 items (MDS-R), to make it applicable to all health professionals over a wide range of clinical contexts [9]. Finally, Epstein et al. [17] revised the MDS-R scale to include other sources of moral distress linked to stress levels related to the team and the health system, as well as to avoid repetition of some of the root causes of distress. 

These researchers devised a 27-item scale (Measure of Moral Distress for Healthcare Professionals, MMD-HP), which could be applied to all healthcare professionals in critical care and long-term intensive care settings. Its reliability and validity were demonstrated in 653 health professionals in the USA, and it has been valued positively by other authors [18], although the need for additional studies to validate MMD-HP in outpatients and long-term acute care has been suggested. MMD-HP has also been recently validated with good results in Japan [19] and in Spain (MMD-HP-SPA) [20].

The aim of the current research was to carry out an advanced analysis of the psychometric properties of the MMD-HP-SPA scale, performing a full validation (using both exploratory and confirmatory factor analysis) of the construct, as well as assessing its internal and external reliability.

## 2. Materials and Methods

### 2.1. Study Design

This was an observational multicentre cross-sectional epidemiological study to discover the psychometric properties of the scale for measuring moral distress in health professionals (MMD-HP-SPA) devised by Rodriguez-Ruiz et al. [20].

A STROBE checklist for cross-sectional studies was followed to enhance methodological rigour.

### 2.2. Participants

The reference population consisted of 45,440 health workers who were doctors or nurses from primary care centres and hospitals, all belonging to the Andalusian Public Health Service.

To calculate the sample size, a minimum sample of 270 participants was considered, according to the method designed by Pérez et al. [21] of selecting 10 subjects for each item on the scale. The study was carried out by convenience sampling of health professionals who answered the online questionnaire in each of the health centres. 

The inclusion criteria were: (a) being a doctor or nurse in the Andalusian Public Health System and belonging to the following services: Primary Care Clinical Management Units (CMU), Palliative Care, Intensive Care (ICU), Internal Medicine, Pneumology, and Accident/Emergency Units (A&E); (b) having signed the informed consent prior to completing the questionnaire. The exclusion criteria included having less than 1 year of experience in practical care work or being a first-year resident/junior doctor in any medical or nursing speciality.

### 2.3. Research Instruments

Resultant variables. Moral distress was measured using the new Spanish version of the MMD-HP-SPA questionnaire, which consists of 27 items scored using a Likert-type scale, with scores ranging from 0 (never) to 4 (very often); and the level of moral suffering with values from 0 (none) to 4 (maximum distress). For each item, the frequency was multiplied by the level of suffering, and the global value of the scale was obtained by adding the scores from all the items, with a final value of 0 to 432 points. The scale provides adimensional values, and it has no cut-off points. 

Sociodemographic variables: age (years), sex, profession, professional experience (years), healthcare setting, and unit.

### 2.4. Procedure

We analysed the psychometric properties of the MMD-HP-SPA scale conducting an advanced analysis of the construct validity and reliability of the scale. Construct validity was studied using both exploratory and confirmatory factor analyses (EFA and CFA) performed on different subgroups within the study sample. For the reliability analysis, we assessed temporal or intra-observer stability by asking 33 participants to complete the questionnaire on two separate occasions separated by an interval of 15 days.

Additionally, we contrasted the following hypotheses based on similar existing studies found in the literature: (a) that among health professionals, nurses would show a higher level of moral suffering than doctors; (b) that health professionals who have considered quitting their job due to moral distress would show a higher level of moral distress.

It was not necessary to analyse inter-observer stability, as this was a self-administered questionnaire. Internal consistency was analysed over the entire study sample.

### 2.5. Ethics

The research study complied with the Declaration of Helsinki and was granted authorization by the Research Ethics Committee (Document N° 5158; 28 September 2021). All the subjects gave their informed consent before participating in the study.

### 2.6. Data Analysis

The quantitative variables were presented by their mean, range, and standard deviation (SD), as well as their median and interquartile range (IQR), while the qualitative variables were represented by their absolute and relative frequency.

The EFA was carried out using the unweighted least squares (ULSs) method to extract factors and the “direct oblimin” oblique rotation method to obtain the rotated matrix [22]. In addition, the KMO (Kaiser–Meyer–Olkin) test was used to estimate the measures’ sampling adequacy, and the Bartlett sphericity test to verify the null hypothesis of the correlation matrix and the sedimentation graph.

For the CFA, the weighted least squares estimator was calculated, and the following goodness-of-fit criteria were applied to the model: root mean square error of approximation (RMSEA) ≤ 0.08, comparative fit index (CFI) ≥ 0.90, and the Tucker–Lewis index (TLI) ≥ 0.90 [23].

Internal consistency or reliability was evaluated using the coefficients Cronbach’s alpha and McDonald’s omega, considering values above 0.70 [24,25] as the reference criterion.

To measure temporal stability (test–retest or intra-observer reliability), we calculated the intraclass correlation coefficient [26].

Student’s *t*-test was used to compare means in independent groups and the ANOVA test was used for 2 or more independent arithmetic means. For the statistical analysis, we used SPSS 22, AMOS-SPSS 24, and EPIDAT 4.2. The level of statistical significance was set at an alpha error of below 5% for all the statistical contrasts and a confidence level of 95% for the confidence intervals [95% CI].

## 3. Results

### 3.1. Characteristics of the Study Sample

A total of 575 health professionals completed the self-administered MMD-HP-SPA questionnaire using an online form. Of the total participants, 392 were female (68.2%) with an overall mean age of 49.1 years (SD = 10.9), 95% CI [48.2–50]. Table 1 shows the main variables of the sample for the total number of subjects and by sample groups used to carry out the EFA (*n* = 300) and CFA (*n* = 275). No significant differences were observed between the samples used for the EFA and CFA, except for the Palliative Care Unit.

Regarding the working variables, 384 of the subjects were doctors (66.8%) and 191 were nurses (33.2%), and the average length of working experience was 17.5 years (SD = 11.1), 95% CI [16.6–18.4]. Of all the services, Primary Care had the highest participation (46.4%) followed by the ICU (17%).

The result of the moral suffering scale (MMD-HP-SPA) was a mean score of 128.5 (SD = 70.9) for the total sample (*N* = 575), with 95% CI [122.7–134.3], a range of 0–424, and a median of 116 and IQR = 97. The accuracy level of the study was 5.8%.

### 3.2. Construct Validity: Hypothesis Testing, Exploratory and Confirmatory Factor Analysis

For the first hypothesis proposed, nursing professionals (*n* = 191) showed a mean value of moral distress of 140.5 (SD = 74.9), while for the medical professionals (*n* = 384), it was 122.5 (SD = 68.1), *T* = 2.89; *p* < 0.01.

For the second hypothesis, a total of 102 professionals stated that they were currently considering quitting their job due to moral distress, with an average score of 177.1 (SD = 75.4), compared to those who stated that they had never thought about changing jobs, 117.7 (SD = 65.2), *T* = −7.37; *p* < 0.001.

The EFA was performed with a subgroup of 300 participants randomly drawn from the total sample (*N* = 575). The sampling adequacy test (KMO) produced a result of 0.925 and the result of the Bartlett sphericity test allowed us to reject the null hypothesis of the correlation matrix (*p* < 0.001).

The rotated components matrix revealed an underlying structure with five factors (Table 2), producing an explained variance of 54.58%. The 27 items of the MMD-HP-SPA questionnaire were included in the five factors and showed moderate-to-high factor loads.

As for the qualitative nature of the factors used, the dimensions addressed by each factor were: Factor 1 “Health care”, Factor 2 “Therapeutic obstinacy-futility”, Factor 3 “Interpersonal relations with the health team”, Factor 4 “External pressure”, and Factor 5 “Concealment of unethical practices”.

**Figure 1:**
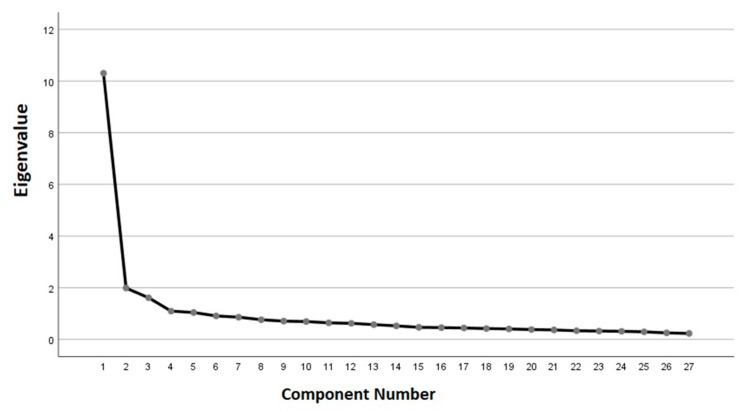
Sedimentation graph for the exploratory factor analysis.

In addition, the sedimentation graph corroborated the five components of the EFA (Figure 1), with the first obtaining an eigenvalue of 10.6 and the value for the last three ranging between 1.6 and 1.17.

Next, a CFA was carried out on a subsample different from the one used in the exploratory study (*n* = 275), randomly selected from the total sample, in order to validate the five-factor structure provided by the EFA.

**Figure 2:**
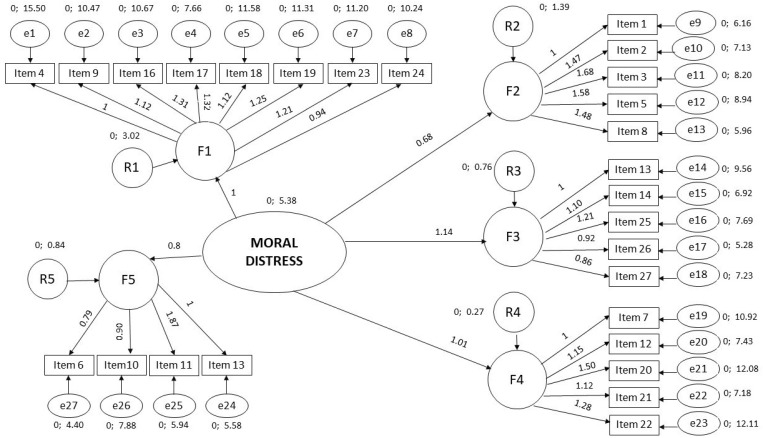
Confirmatory factor analysis (*n* = 275).

The first model obtained a value of χ^2^ = 1033.9, where, in the pairwise parameter comparison analysis, it was considered advisable to make some changes, such as moving item 25 from Factor 1 to Factor 3, to improve the adjustment of the second model, which produced χ^2^ = 972.4. Figure 2 shows the final model. In addition, Table 3 shows the goodness of fit indicators of models 1 and 2 of the CFA.

### 3.3. Internal Reliability and Temporary Reliability

The internal reliability (consistency) of the MMD-HP-SPA version, analysed by Cronbach’s alpha and McDonald’s omega statistics, produced a value over 0.94. Table 4 shows the internal consistency results for the total sample, as well as for the five factors revealed through the CFA, with all the values above 0.7 (acceptable internal consistency).

Finally, we performed the analysis of temporal reliability or intra-observer stability (test–retest) in a sample of 33 participants who completed the questionnaire on two separate occasions and 15 days apart. The intraclass correlation coefficient was 0.993, 95% CI [0.987–0.997], confirming excellent temporal reliability.

To measure the concordance of the scores before and after, we noted that almost 100% of the pairs of scores were between the mean ± 1.96 SD.

## 4. Discussion

In this study, we assessed the psychometric properties of internal validity and external reliability for the MMD-HP-SPA questionnaire devised by Rodriguez-Ruiz et al. (Spanish version of the MMD-HP questionnaire) [20]. To achieve this, a questionnaire was completed by 575 health professionals (doctors and nurses) belonging to different hospital services (ICU, Internal Medicine, Palliative Care, Pulmonology) and community services (Health Centres, A&E), all from the Andalusian Public Health Service (Spain).

The level of moral distress found in the study sample reached a mean = 128.5 (SD = 70.9) and median = 116 (IQR = 97), notably higher than in another sample of 1065 intensive care health workers from Spain [27] with a median = 68 (IQR = 96), possibly due to the accumulated burnout from a year and a half of COVID-19 pandemic. These results are also higher than the levels reported in Japan in a sample of 308 hospital health professionals, with a mean value of moral distress of 98.2 (SD = 60) [19], but more in line with the results obtained by Epstein et al. [17], who found a mean of 108.9 (SD = 70.7) in 653 USA professionals.

The level of moral distress found in the study sample reached a mean = 128.5 (SD = 70.9) and median = 116 (IQR = 97), notably higher than in another sample of 1065 intensive care health workers from Spain [27] with a median = 68 (IQR = 96), possibly due to the accumulated burnout from a year and a half of COVID-19 pandemic. These results are also higher than the levels reported in Japan in a sample of 308 hospital health professionals, with a mean value of moral distress of 98.2 (SD = 60) [19], but more in line with the results obtained by Epstein et al. [17], who found a mean of 108.9 (SD = 70.7) in 653 USA professionals.

Regarding the construct validation of the scale, Rodriguez-Ruiz et al. [20], in their validation of the Spanish version of the questionnaire (MMD-HP-SPA), carried out a descriptive study with 1,115 health professionals (doctors and nurses) working at ICUs in Galicia (Spain). To analyse the psychometric properties, they performed an EFA, but did not follow it up with a CFA. The principal component analysis in the EFA used the extraction method, setting the eigenvalue criterion ≥ 1 to extract the component, and a rotated solution using the Varimax method.

These researchers obtained a factorial model with four components, which accounted for 60% of its variance. The factors were: (i) patient-related causes: (ii) clinical causes; (iii) witnessing unethical behaviour, as well as the breakdown of the interaction between the team–patient–family; (iv) feeling the integrity of a member of the team is in danger or feeling intimidated or insecure.

This underlying structure is similar to that used in the original questionnaire by Epstein et al. [17], with four factors covering the main causes of moral distress: (i) the health system; (ii) patient–clinic relations; (iii) equipment and personal danger or vulnerability; (iv) interactions between the team and the patient.

In contrast, in this study, to measure the construct validity we first performed an EFA, followed by a CFA to corroborate the former. The EFA was therefore carried out on a subsample of 300 participants using a different methodology (unweighted least squares method and oblique rotation with oblimin direct) in relation to Rodriguez-Ruiz et al. [20] for the Spanish version of the MMD-HP, followed by a CFA on the remaining 275 subjects from the study sample to ratify the five-factor model obtained in the EFA, which accounted for up to 54.58% of its variance. The five components of the model were: (i) healthcare; (ii) therapeutic obstinacy–futility; (iii) interpersonal relationships with health team; (iv) external pressure; (v) concealing unethical practices. 

Comparing our results with those reported by Rodriguez-Ruiz et al. [20], their “patient” factor corresponds to our factor labelled “therapeutic obstinacy-futility”, the “clinical” factor coincides with our “health care”, and “the integrity of a member of the team is in danger or feels intimidated or insecure” correlates with “external pressure” in our model. The main difference with the model by Rodriguez-Ruiz et al. [20] lies in the subdivision of Factor 3, namely “witnessing unethical behaviour, including the breakdown of interaction between team-patient-family”, into two factors in our model, namely Factor (iii) “concealing unethical practices” and Factor (v) “interpersonal relationships with the health team”.

Unfortunately, very few validations of the MMD-HP scale devised by Epstein et al. [17] exist in other countries. Fischer-Grönlund et al. [28] produced a translation and cultural adaptation of the MMD-HP scale but did not analyse its construct validity. In addition, Fujii et al. [19] performed a validation of this scale in Japan with 385 health professionals and reported results on the construct validity using EFA and CFA, producing a factorial model of four factors which accounted for 41% of its variance: (i) clinical issues; (ii) health system; (iii) interpersonal relationships within the health team; (iv) relationships between the healthcare team and the patient. They reported a goodness of fit for the model after the CFA which was slightly better than that found in our present study; CFI: 0.91 vs. 0.844; RMSEA: 0.061 vs. 0.086; and finally, χ^2^: 891 vs. 972.

Furthermore, the construct validity was complemented by a contrast of previous hypotheses drawn from the results obtained in each study. In our research, the hypothesis that nurses showed greater moral distress than doctors (140.5 vs. 122.5) was verified, as reported by Epstein et al. [17] (112.3 vs. 96.2). However, Rodriguez-Ruiz et al. [20] obtained a higher level of moral distress in doctors in intensive care (61 vs. 80), in line with the results in Japan (97.2 vs. 104) [19]. Similarly, health professionals who stated that they were currently thinking of quitting their job at the time of the survey presented significantly higher levels of moral distress.

Regarding internal reliability, Epstein et al. [17], in the original MMD-HP questionnaire, showed a value of Cronbach’s α = 0.93, while Fujii et al. [19] found an internal consistency of Cronbach’s α = 0.91. In their study, Rodriguez-Ruiz et al. [27] reported a high global internal consistency (Cronbach’s α = 0.94 and alpha–ordinal = 0.98), similar to the internal consistency found in our research (Cronbach’s α = 0.94 and McDonald’s ω = 0.95). However, Rodriguez-Ruiz et al. [27] did not include internal consistency results for each of the four components produced by the EFA. Our study showed that the five factors of the questionnaire obtained an acceptable internal reliability, with Cronbach’s α and McDonald’s ω values over 0.7. Finally, the temporal reliability of the MMD-HP-SPA scale was excellent, as shown by the agreement of nearly 1 (ICC = 0.993). In summary, the studies cited in this discussion did not provide results for temporal stability which could be compared with ours.

### Strengths and Limitations

The following are some of the key strengths of our study: (i) obtaining a large study sample (*N* = 575) which included health professionals from various contexts (hospitals and community health centres) and different hospital services, as was the original objective of the MMD-HP questionnaire devised by Epstein et al. [17]; (ii) the large sample size also helped to improve the accuracy of the research (5.7%); (iii) dividing the participants into two subgroups to carry out an independent confirmatory factor analysis which reinforced the confirmatory results; (iv) the optimal temporal stability and internal reliability of the scale, both globally and for the latent dimensions.

However, it also has its limitations, such as the use of an online questionnaire for data collection, which could limit the participation in some working sectors. This circumstance also meant that we were unable to calculate the response rate, as we did not know how many health professionals had accessed the questionnaire link. Second, the lack of validation studies from other countries made it difficult to test the underlying structure of the factor analysis.

## 5. Conclusions

A reliable and validated instrument is needed to measure the moral distress of professionals in the health system. If this condition is not adequately detected and managed, it can lead to stronger reactions that may compromise the long-term continuity of the professionals in their jobs. Awareness of the actual situation could promote the implementation of coping measures to improve the health of nurses and doctors and, consequently, the quality of patient care.

In conclusion, we can confidently state that, after detailing and testing the psychometric properties of the MMD-HP-SPA scale using an advanced analysis, our research shows that it has solid construct validity, excellent internal consistency, optimal temporal reliability, and underlying dimensions which effectively explore the causes of moral suffering among health professionals. The internal and external validity of the MMD-HP-SPA scale guarantees its use as a measure of moral distress in health professionals in Spain in hospital and community settings.

## Figures and Tables

**Table 1:** Characteristics of the study sample.

Variable	Total *N* = 575	EFA ^1^ *n* = 300	CFA ^2^ *n* = 275	*p*
Age	49.1 (10.9)	48.7 (11.2)	49.5 (10.6)	0.35
Male	183 (31.8%)	97 (32.3%)	86 (31.3%)	0.79
Female	392 (68.2%)	203 (67.7%)	189 (68.7%)	
Doctors	384 (66.8%)	200 (66.7%)	184 (67.3%)	0.58
Nurses	191 (33.2%)	100 (33.3%)	91 (32.7%)	
Experience	17.5 (11.1)	17.1 (10.8)	17.9 (11.4)	0.44
Primary Care	262 (46.4%)	125 (41.7%)	137 (49.8%)	0.06
Palliative Care	36 (6.4%)	25 (8.3%)	11 (4%)	0.05
Internal Medicine	58 (10.3%)	32 (10.7%)	26 (9.5%)	0.73
Pneumology	50 (8.9%)	27 (9%)	23 (8.3%)	0.9
ICU ^3^	96 (17%)	51 (17%)	45 (16.4%)	0.92
A&E ^4^	62 (11%)	32 (10.7%)	30 (10.9%)	0.96

^1^ EFA: exploratory factor analysis; ^2^ CFA: confirmatory factor analysis; ^3^ ICU: intensive care unit; ^4^ A&E: accident/emergency unit.

**Table 2:** Exploratory factor analysis (*n* = 300). Rotated factor matrix.

	Factors				
	1	2	3	4	5
Item_4	**0.559**	−0.115	−0.008	0.213	0.187
Item_9	**0.622**	0.081	0.035	−0.112	0.177
Item_16	**0.761**	0.132	0.063	−0.005	−0.232
Item_17	**0.877**	−0.008	0.031	−0.066	−0.082
Item_18	**0.721**	−0.026	0.093	−0.016	0.082
Item_19	**0.697**	−0.018	0.021	0.096	0.025
Item_23	**0.508**	0.055	−0.023	0.315	0.126
Item_24	**0.446**	0.063	0.146	0.014	0.317
Item_25	**0.296**	−0.100	0.318	0.278	0.098
Item_1	0.061	**0.341**	0.010	−0.214	0.197
Item_2	0.032	**0.762**	−0.001	0.004	0.003
Item_3	0.207	**0.593**	0.051	0.173	−0.044
Item_5	−0.042	**0.810**	0.043	0.006	−0.039
Item_8	−0.119	**0.607**	0.117	0.132	0.238
Item_13	0.203	0.167	**0.529**	−0.038	−0.085
Item_14	0.223	0.158	**0.601**	−0.027	−0.052
Item_26	0.000	0.104	**0.746**	0.005	0.005
Item_27	−0.032	−0.122	**0.763**	0.078	0.064
Item_7	0.235	0.178	0.052	**0.272**	0.145
Item_12	0.177	0.231	0.007	**0.440**	0.087
Item_20	0.118	−0.010	0.200	**0.528**	0.067
Item_21	−0.084	0.142	0.408	**0.430**	0.063
Item_22	0.297	0.286	0.164	**0.331**	−0.066
Item_6	0.015	0.162	−0.022	0.106	**0.563**
Item_10	0.188	0.197	−0.014	0.106	**0.339**
Item_11	0.212	−0.108	0.311	0.145	**0.384**
Item_15	0.010	0.021	0.325	−0.113	**0.409**

Extraction method: unweighted least squares (ULSs), Rotation method: direct oblimin with Kaiser normalisation.

**Table 3:** Indicators of goodness of fit for the models obtained by confirmatory factor analysis.

	CMIN ^1^	RMSEA ^2^	CFI ^3^	TLI ^4^	NFI ^5^	P-RAT ^6^	PNFI ^7^	PCFI ^8^	AIC ^9^
Model 1	<0.05	0.9	0.829	0.81	0.772	0.909	0.701	0.753	1205.9
Model 2	<0.05	0.86	0.844	0.828	0.785	0.909	0.714	0.767	1144.3

^1^ CMIN: Chi^2^ fit index; ^2^ RMSEA: root mean square error of approximation; ^3^ CFI: comparative fit index; ^4^ TLI: Tucker–Lewis index; ^5^ NFI: normal fit index; ^6^ P-RAT: parsimony ratio; ^7^ PNFI: product of P-Ratio by NFI; ^8^ PCFI: product of P-Ratio by CFI; ^9^ AIC: Akaike information criterion.

**Table 4:** Internal reliability (consistency) analysis for the Spanish version of the MMD-HP-SPA.

Indicator	Total *N* = 575	Factor 1	Factor 2	Factor 3	Factor 4	Factor 5
Cronbach’s alpha	0.94	0.892	0.832	0.833	0.779	0.741
McDonald’s omega	0.945	0.894	0.843	0.838	0.785	0.734

## Data Availability

The datasets used and analysed during the current study are available from the corresponding author upon reasonable request after approval from all the authors.
